# Crystal structures, phase transitions, thermodynamics, and molecular dynamics of organic–inorganic hybrid crystal [NH(CH_3_)_3_]_2_ZnCl_4_

**DOI:** 10.1038/s41598-024-53965-6

**Published:** 2024-02-11

**Authors:** A Young Kim, Changyub Na, Ae Ran Lim

**Affiliations:** 1 Graduate School of Carbon Convergence Engineering, Jeonju University, Jeonju, 55069 South Korea; 2 Department of Science Education, Jeonju University, Jeonju, 55069 South Korea

**Keywords:** Structural materials, Materials science, Chemistry, Organic chemistry

## Abstract

Understanding the physical properties of organic–inorganic hybrid [NH(CH_3_)_3_]_2_ZnCl_4_ is necessary for its potential application in batteries and fuel cells due to its environmentally-friendly, and highly stable character. Here, we determine its overall properties in detail, such as its orthorhombic crystal structure, and phase transition temperatures associated with five different phases. Structural geometry was studied by the chemical shifts caused by the local field around ^1^H. No changes were observed for the environment around ^1^H for CH_3_, whereas the ^1^H chemical shifts around NH in the cation were shown due to the change in the hydrogen bond N‒H···Cl. This is related to the change in Cl around Zn in the anion. In addition, the coordination geometry of ^14^N and ^1^H around ^13^C exhibited increased symmetry at high temperatures. Finally, we were able to understand its molecular dynamics by the significant change with temperature observed from the spin–lattice relaxation time T_1ρ_ values, which represent the energy transfer for the ^1^H and ^13^C atoms of the cation. The activation energies obtained from the T_1ρ_ results were 3–4 times large at phase I (> 348 K) than at phase V and IV (< 286 K). The relaxations show that the energy barriers in phases IV and V are related to the reorientation of methyl groups around the triple symmetry axis, while the reorientation of methyl groups of the cation in phase I is related to as a whole.

## Introduction

Organic–inorganic hybrid compounds have been a field of great interest recently because of their potential applications. Their potential applications were mainly related to sensors, fuel cells, solar cells, light emitting transistor, and optical switches^1–5^. Recently, CH_3_NH_3_Pb*X*_3_ (*X* = Cl, Br, I) has been used for solar cells^6–8^, but these materials were easily degraded in humid air and toxic due to the presence of Pb. Therefore, it is necessary to develop highly stable, eco-friendly hybrid perovskite solar cells. A new type of perovskite compound [(CH_3_)_2_NH_2_]Zn(HCOOO)_3_, consisting of a cation and a metal ion connected by formate ions, has been reported^9–12^. Especially, research on [NH_3_(CH_2_)_*n*_NH_3_]*MX*_4_ (*n*=2, 3, 4, …, *M* represents divalent metals; Mn, Co, Cu, Zn, Cd, Pb, and *X* halogens; Cl, Br, I), with a focus on the physicochemical properties of their perovskite structure and dynamics investigation, has been garnering much attention^13–16^. As a new alternative, compounds of [NH(CH_3_)_3_]_2_*MX*_4_ may be described as sequences of alternating organic–inorganic layers^17–23^. For these organic-inorganic materials, the ammonium ion of the organic group forms the N−H···X hydrogen bond with the halide ion of the inorganic layer. The individual *MX*_4_ tetrahedral anions in these materials were completely isolated and surrounded by organic [NH(CH_3_)_3_] cations. These substances were also expected to act as potential proton conductors owing to the existence of hydrogen bonds^24,25^.

Solid-state nuclear magnetic resonance (NMR) provides information for studying local structure and mobility. By considering the temperature dependence of spin-lattice relaxation time of each nucleus, information can be obtained on the dynamical motion occurring in different environments^26–33^. Solid-state NMR on hybrid perovskite materials has also garnered considerable attention.

According to the previously reported, the phase transition temperatures T_C_ of [NH(CH_3_)_3_]_2_ZnCl_4_ crystal, trimethylammonium tetrachlorozincates, were reported by Kapustianik et al.^34^ at 276.5 K and 310 K. According to Sveleba et al.^35^, it was reported that the phase transition temperatures by dielectric properties occur at 269, 282, and 309 K. After that, the T_C_ of [NH(CH_3_)_3_]_2_ZnCl_4_ defined by differential scanning calorimetry (DSC) has five phase transitions at 255 K, 282 K, 302 K, 320 K, and 346 K^19^. The [NH(CH_3_)_3_]_2_ZnCl_4_ crystal at 300 K has an orthorhombic structure with the space group *Pnma*, and its lattice constants are *a*=10.660 Å, *b*=9.629 Å, *c*=14.991 Å. The [NH(CH_3_)_3_]_2_ZnCl_4_ structure consists of trimethylammonium [NH(CH_3_)_3_] cation and [ZnCl_4_] tetrahedron. This crystal is characterized by hydrogen bonds N−H···Cl connecting the organic cation to the inorganic anion. And, the ferroelectric properties and characterization of phase transitions were discussed by Raman spectroscopy^36^.

In this study, the [NH(CH_3_)_3_]_2_ZnCl_4_ single crystals were grown using the aqueous solution method, and their structures, the phase transition temperatures, and thermal properties were investigated. Nuclear magnetic resonance (NMR) chemical shifts as a function of temperature were measured to investigate the coordination geometry for the ^1^H and ^13^C atoms in the cations of this crystal. From these results, the N‒H···Cl hydrogen bond according to the ligands between the cations and anions was considered. And, the spin-lattice relaxation times T_1ρ_ representing the energy transfer around the ^1^H and ^13^C atoms were discussed as a function of temperature, and their activation energies E_a_ are determined. The results of the single-crystal structure, NMR chemical shifts, and T_1ρ_ are predicted important information on the crystal configuration and the energy transfer mechanism for the potential applications.

## Methods

### Crystal growth

Single crystals of [NH(CH_3_)_3_]_2_ZnCl_4_ were prepared from [NH(CH_3_)_3_]Cl (Aldrich, 98%) and ZnCl_2_ (Aldrich, 98%) in a ratio of 2:1. The mixed compounds were heated to make a homogeneous solution. The mixed solution was filtered once through filter paper, and colorless single crystals grown by slow evaporation were obtained after few days in a thermostat of 300 K.

### Characterization

The lattice parameters of [NH(CH_3_)_3_]_2_ZnCl_4_ crystals were determined by single-crystal X-ray diffraction (SCXRD) at the Seoul Western Center of the Korea Basic Science Institute (KBSI). The crystal block was mounted on diffractometer (Bruker D8 Venture PHOTON III M14) equipped with a graphite-monochromated Mo-Kα (λ=0.71073 Å) radiation source. Data using SMART APEX3 and SAINT was collected and integrated. The structure was solved using direct methods and refined by full matrix least-squares on F2 using SHELXTL^37^. Additionally the powder XRD (PXRD) patterns of the [NH(CH_3_)_3_]_2_ZnCl_4_ crystals were measured using an XRD system with the same Mo-Kα used in SCXRD.

DSC measurement was performed using a DSC instrument (TA Instruments, DSC 25) with a heating speed of 10 °C/min between the 200 and 573 K temperature range under a flow of nitrogen gas. The changes of the single crystal according to temperature change were measured using an optical polarizing microscope. A hot stage was used to change the temperature (Linkam THMS 600).

Thermogravimetric analysis (TGA) was also measured with a heating speed of 10 °C/min in the temperature range between 300 and 873 K under nitrogen gas.

The magic angle spinning (MAS) NMR chemical shifts and the spin-lattice relaxation time T_1ρ_ of the [NH(CH_3_)_3_]_2_ZnCl_4_ crystals were measured using a solid-state NMR spectrometer (Bruker, AVANCE III+) at the same facility, Western Seoul Center of the KBSI. The Larmor frequency for ^1^H NMR experiment was 400.13 MHz, and that for the ^13^C NMR experiment was 100.61 MHz. The sample was placed in cylindrical zirconia rotors and measured with a spin speed of 10 kHz for the MAS NMR measurements, in order to the reduce the spinning sideband. Chemical shifts were referenced to adamantane and tetramethylsilane (TMS) for ^1^H and ^13^C, respectively, as standard materials in order to accurate the chemical shift measurements. 1D NMR spectrum for ^1^H and ^13^C was performed in the delay time of 2–20 s. The ^1^H T_1ρ_ values were measured using π/2−*τ* spin-lock pulse for a duration of *τ*, and the π/2 pulse width was 3.65–4 μs. And, the ^13^C T_1ρ_ values were obtained by varying the duration of a ^13^C spin-locking pulse after the CP preparation period. The ^13^C T_1ρ_ values were obtained using CP-*τ* acquisition.

## Experimental results

### FT-IR spectra

**Figure 1:**
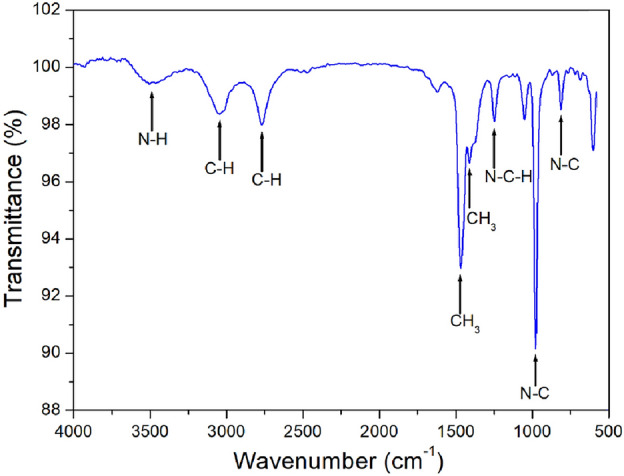
FT-IR spectrum of [NH(CH_3_)_3_]_2_ZnCl_4_ at room temperature.

The FT-IR spectrum at 300 K was recorded within the 4000–500 cm^−1^ range. The result is shown in Fig. 1, and the peaks near 813 and 978 cm^−1^ are assigned to the N–C mode. And, the peak at 1253 cm^−1^ is related to the deformation vibration N–C–H. The bands observed 1410 and 1468 cm^−1^ are assigned to the CH_3_ mode. The bands near 2769 and 3058 cm^−1^ are related to the stretching mode C–H, and the peak at 3493 cm^–1^ is the N–H stretching mode. This result agrees well the previously reported results of [NH(CH_3_)_3_]_2_CdCl_4_^23^.

### Crystal structure

**Figure 2:**
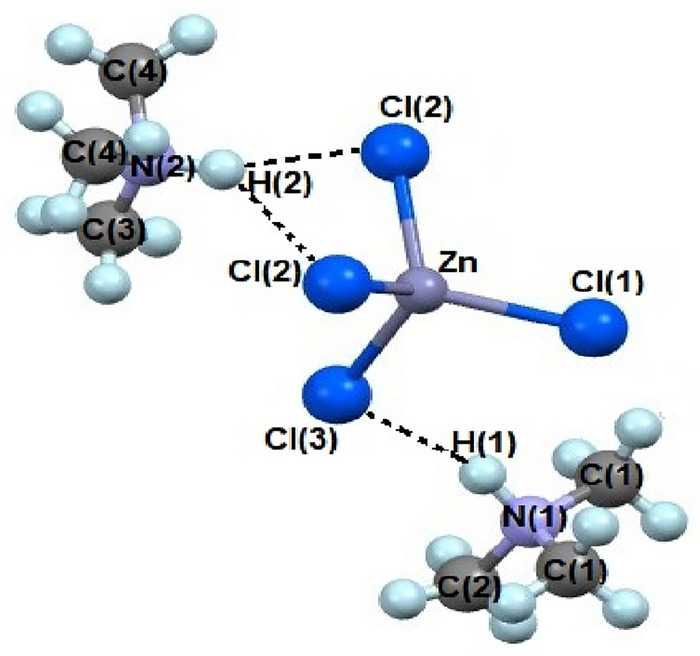
Crystal structure of [NH(CH_3_)_3_]_2_ZnCl_4_ at 300 K.

Single crystal XRD results for the [NH(CH_3_)_3_]_2_ZnCl_4_ crystal grown here were obtained at 300 K. The single crystal structure has an orthorhombic system with space group *Pnma*, lattice constants a = 10.6279(4) Å, b = 9.6297(4) Å, c = 14.9880(7) Å, and Z = 4, which are consistent with previously reported results^19^. Figure 2 shows the thermal ellipsoid and atomic number for each atom, and XRD data of [NH(CH_3_)_3_]_2_ZnCl_4_ crystals are shown in Table 1. The infinite chains consisted of face-shared ZnCl_4_ tetrahedra and four doubly bridging Cl^−^ ions linked to adjacent Zn centers. As shown in Fig. 2, this compound is connected by three hydrogen bond N‒H···Cl between the [NH(CH_3_)_3_] cation and the [ZnCl_4_] anion. The bond-lengths for Zn‒Cl, N‒C, N‒H···Cl and bond-angles for Cl‒Zn‒Cl and N‒H···Cl are shown in Table 2. Here, the N‒H···Cl hydrogen bond consists of an angle greater than 120°.

**Table 1:** Crystal data and structure refinement for [NH(CH_3_)_3_]_2_ZnCl_4_ at 300 K.

Chemical formula	C_6_H_20_N_2_ZnCl_4_
Weight	327.41
Crystal system	Orthorhombic
Space group	*Pnma*
T (K)	300
*a* (Å)	10.6279 (4)
*b* (Å)	9.6297 (4)
*c* (Å) α (°) β (°) γ (°)	14.9880 (7) 90 90 90
Z	4
V (Å^3^)	1533.92
Radiation type	Mo-Kα
Wavelength (Å)	0.71073
Reflections collected	12,566
Independent reflections	2009 (*R*_int_ = 0.0333)
Goodness-of-fit on *F*^2^	1.037
Final *R* indices [I > 2sigma(I)]	*R*_1_ = 0.0485, *wR*_2_ = 0.1355
*R* indices (all data)	*R*_1_ = 0.0751, *wR*_2_ = 0.1559

**Table 2:** Bond-lengths (Å) and bond-angles (°) for [NH(CH_3_)_3_]_2_ZnCl_4_ at 300 K.

Zn-Cl(1)	2.2510 (7)
Zn-Cl(2)	2.2577 (11)
Zn-Cl(2)#1	2.2577 (11)
Zn-Cl(3)	2.2855 (18)
N(1)-C(1)#1	1.433 (7)
N(1)-C(1)	1.433 (7)
N(1)-C(2)	1.471 (9)
N(2)-C(3)	1.377 (17)
N(2)-C(4)	1.442 (8)
N(2)-C(4)#1	1.442 (8)
N(1)-H(1)	0.9800
N(2)-H(2)	0.9800
C(1)-H(1)	0.9600
C(2)-H(2)	0.9600
C(3)-H(3)	0.9600
C(4)-H(4)	0.9600
N(1)-H(1)-Cl(3)	3.185
N(2)-H(2)-Cl(2)	3.627
N(2)-H(2)-Cl(2)#1	3.627
Cl(1)-Zn-Cl(2)	113.04 (5)
Cl(1)-Zn-Cl(2)#1	113.04 (5)
Cl(2)-Zn-Cl(2)#1	104.48 (7)
Cl(1)-Zn-Cl(3)	108.68 (7)
Cl(2)-Zn-Cl(3)	108.71 (5)
Cl(2)#1-Zn-Cl(3)	108.71 (5)
N(1)-H(1)-Cl(3)	160.96
N(2)-H(2)-Cl(2)	135.05

### Phase transition temperatures

**Figure 3:**
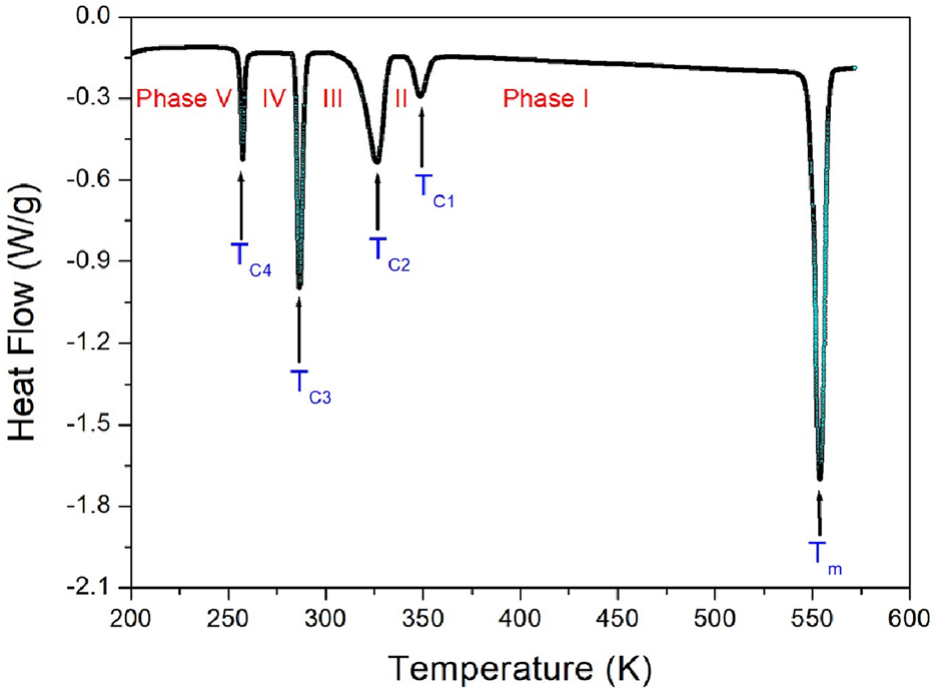
Differential scanning calorimetry curve of [NH(CH_3_)_3_]_2_ZnCl_4_ measured at a heating rate of 10 °C/min.

The DSC thermogram for powder [NH(CH_3_)_3_]_2_ZnCl_4_ was measured in the temperature range from 200 to 573 K with the heating rate of 10 ℃/min. Figure 3 shows the four endothermic peaks at 257 K, 286 K, 326 K, and 348 K. And, the one strong endothermic peak at 553 K was obtained. The enthalpies for the five peaks were 2.54, 7.99, 7.53, 1.86, and 17.58 kJ/mol, respectively. Starting from 200 K, these five phases were denoted as phase V below 257 K, phase IV between 257 and 286 K, phase III between 286 and 326 K, phase II between 326 and 348 K, and phase I above 348 K, shown in Fig. 3.

In order to know whether the five endothermic peaks shown in the DSC results shown in Fig. 3 are the phase transition temperatures or melting temperature, the changes of a single crystal were observed using an optical polarizing microscope with increasing temperature. Until the temperature rises to 530 K, the single crystal is almost unchanged, but the single crystal began to melt above 550 K.

**Figure 4:**
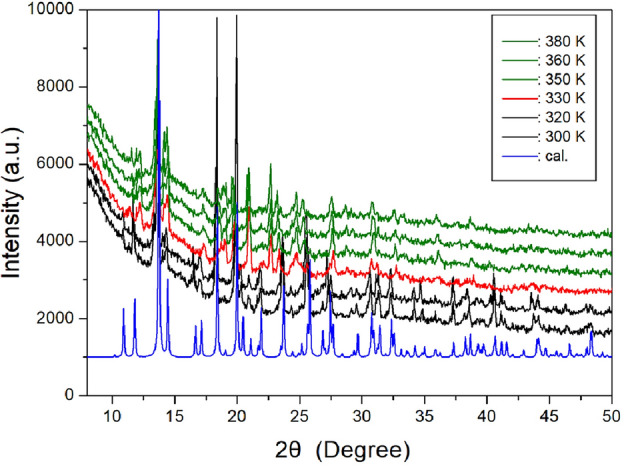
Powder X-ray diffraction patterns of [NH(CH_3_)_3_]_2_ZnCl_4_ at phases III, II, and I. The blue colour is the theoretical powder pattern at 300 K.

In addition, a powder XRD experiment was measured according to the temperature change. The PXRD patterns in the range of 8°–50° (2θ) are shown at various temperature in Fig. 4. The PXRD patterns below 330 K (black) differ from that recorded above 330 K (red); this difference is related to the structural phase transition in T_C2_ (= 326 K). Furthermore, the XRD patterns recorded above 330 K differed from those recorded at 350 K (olive), and this difference is related to the phase transition in T_C1_ (= 348 K). These results are consistent with those of the DSC result. And, the theoretical XRD pattern at 300 K, which agrees well with the experimental pattern, is shown in Fig. 4.

The phase transition temperatures and melting temperature shown in the PXRD and polarizing microscope results are agree well with the endothermic peaks obtained in the DSC curve. From the DSC, PXRD, and optical polarizing microscopy results, the phase transition temperatures were determined as T_C4_ = 257 K, T_C3_ = 286 K, T_C2_ = 326 K, T_C1_ = 348 K, and the melting temperature was T_m_ = 553 K.

### Thermal property

**Figure 5:**
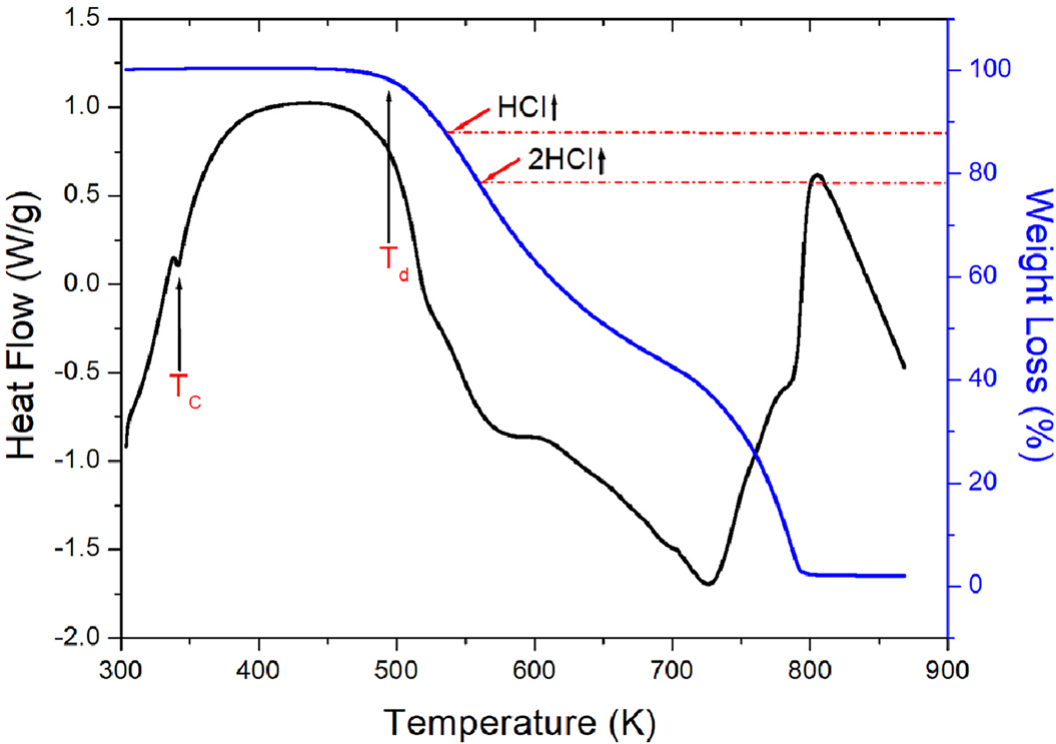
Thermogravimetric analysis and differential thermal analysis curves of [NH(CH_3_)_3_]_2_ZnCl_4_.

TGA curves shown in Fig. 5 were obtained with the increasing temperature. In the TGA curve, the partial decomposition temperature T_d_ representing a weight loss of 2 % was 495 K, and this material was thermal stable up to 495 K. The molecular weight of the [NH(CH_3_)_3_]_2_ZnCl_4_ crystal as the temperature increased was abruptly decreased by the partial decomposition. The molecular weight losses of 11 % and 22 % calculated from the total molecular weight were by the partial decomposition of HCl and 2HCl, respectively. The initial weight loss (45 %) was occurred in the range of 500–630 K. On the other hand, an endothermic peak at 342 K appeared in the differential thermal analysis (DTA) curve, which is shown as a differential form of TGA, was in good agreement with the phase transition temperature T_C1_ shown in the DSC result. In addition, it was found that total weight loss occurred at temperatures near 800 K.

### ^1^H and ^13^C MAS NMR chemical shifts

**Figure 6:**
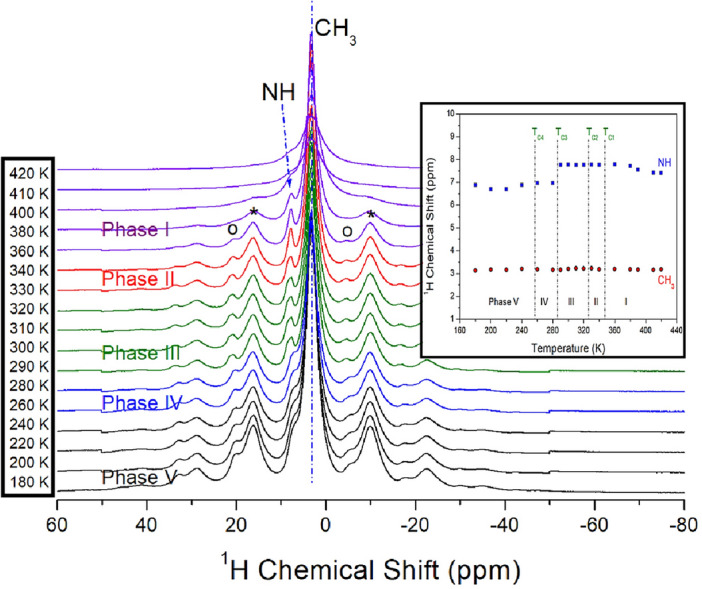
^1^H MAS NMR chemical shifts of NH and CH_3_ in [NH(CH_3_)_3_]_2_ZnCl_4_ at phases V, IV, III, II, and I. The open circles are sideband for NH, and the asterisks are is sideband for CH_3_ (inset: the ^1^H chemical shifts for NH and CH_3_ near phase transition temperatures).

The NMR chemical shifts for ^1^H in the [NH(CH_3_)_3_]_2_ZnCl_4_ crystal were recorded at phases V, IV, III, II, and I, as shown in Fig. 6. The ^1^H NMR spectra for NH and CH_3_ were obtained, and their sidebands for ^1^H spectrum were represented as the open circles and asterisks, respectively. At 300 K, the ^1^H chemical shift for NH was recorded about 7.78 ppm and the ^1^H chemical shift for CH_3_ was obtained about 3.21 ppm. Depending on the temperature change, there is no change near T_C4_, T_C2_, and T_C1_, but the ^1^H chemical shift for NH near T_C3_ shows a change. As shown inside of Fig. 6, the ^1^H chemical shifts for CH_3_ were hardly change as the temperature increased, whereas the ^1^H chemical shifts for NH were changed, and this result means that the coordination geometry around ^1^H for CH_3_ does not changes according to the temperature change, whereas the coordination geometry around ^1^H for NH was changes.

**Figure 7:**
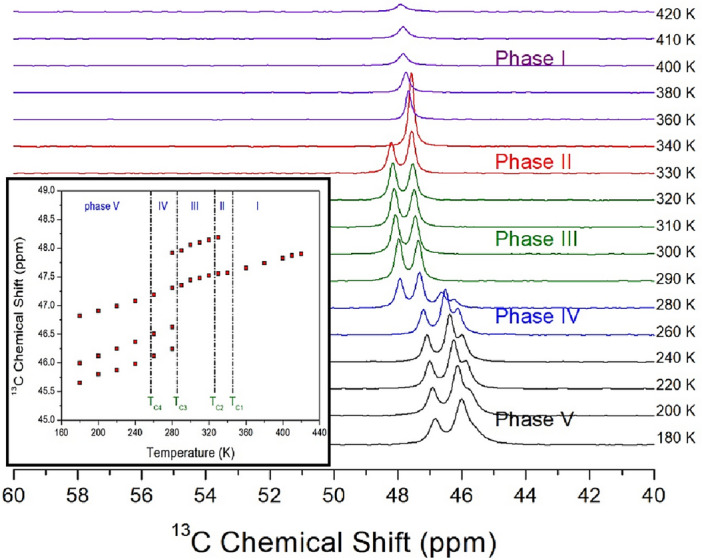
^13^C MAS NMR chemical shifts in [NH(CH_3_)_3_]_2_ZnCl_4_ at phases V, IV, III, II, and I (inset: the ^13^C chemical shifts for CH_3_ as a function of temperature).

The ^13^C NMR chemical shifts of [NH(CH_3_)_3_]_2_ZnCl_4_ were measured in phases V, IV, III, II, and I with increasing temperature as shown in Fig. 7. In the structure of the crystal shown in Fig. 2, three ^13^C atoms are bonded with ^1^H and ^14^N. The ^13^C chemical shifts for CH_3_ at 220 K of phase V showed three signals (46.98, 46.24, and 45.87 ppm), it reduced to two signals at 300 K of phase III (48.05 and 47.44 ppm), and it also reduced to one signal at 420 K of phase I (47.89 ppm). Although ^14^N and ^1^H are bonded around ^13^C in the cation, the environments around ^13^C can be different depending on the nearby Cl^−^. That is, the change of ^13^C chemical shifts was not seen near T_C4_, whereas the change of chemical shift was large in T_C3_. In addition, two signals were obtained between T_C3_ and T_C2_, and at the temperature above T_C1_, only one signal was obtained. The change in the number of ^13^C chemical shifts can be explained as follows. That is, in phases V and IV, there are three CH_3_ with different environments, and in phases III and II, there are two CH_3_ with different environments. In particular, in phase I, it was found that all the environments around ^13^C in CH_3_ were the same. The number of peaks in the ^13^C NMR spectra decreases with increasing temperature, indicating an increase in coordination symmetry around the [NH(CH_3_)_3_] cations.

### ^1^H and ^13^C NMR spin-lattice relaxation times

**Figure 8:**
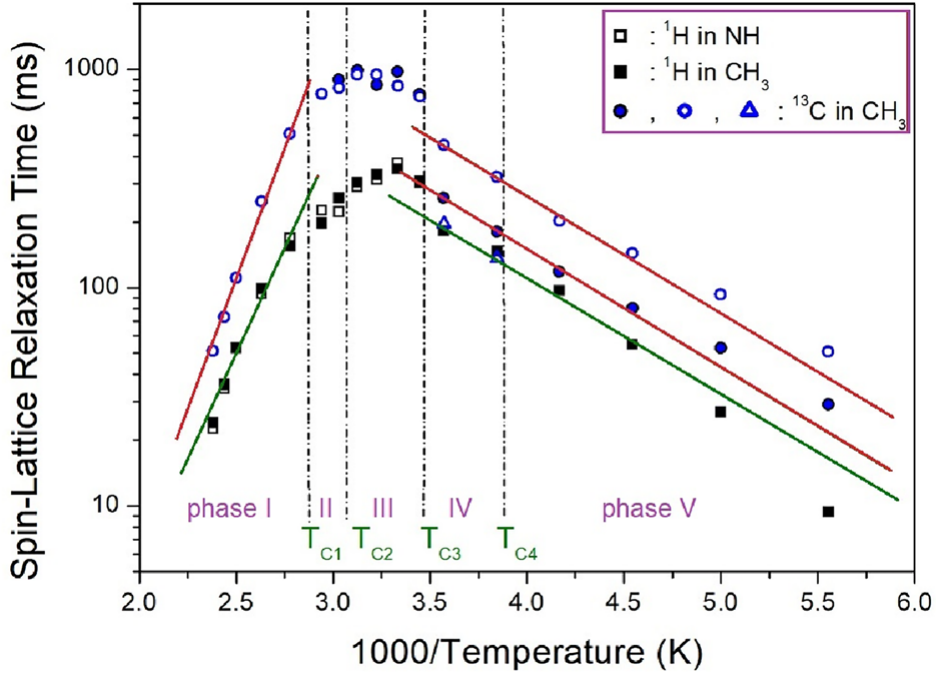
^1^H and ^13^C NMR spin–lattice relaxation times T_1ρ_ in [NH(CH_3_)_3_]_2_ZnCl_4_ at phases V, IV, III, II, and I. The slopes of lines are represented the activation energies by the T_1ρ_ as a function of inverse temperature.

To understand the spin-lattice relaxation time T_1ρ_, the signal intensities of ^1^H and ^13^C NMR spectra were measured according to the change of the delay times. The decay curves by the change in the intensities and delay times are represented as following equation^38–40^:

1 $$P\left( t \right) \, = \, P\left( 0 \right)\exp ( - t/T_{1\rho } ),$$

where P(*t*) is the intensity of the spectrum at time *t* and P(0) is the intensity of the spectrum at time *t* = 0. The T_1ρ_ values for ^1^H and ^13^C in [NH(CH_3_)_3_]_2_ZnCl_4_ are obtained using Eq. (1), and their results are represented in Fig. 8 at phases V, IV, III, II, and I. ^1^H and ^13^C T_1ρ_ at phases V and IV increases as the temperature increases and show a maximum value near 300 K. The T_1ρ_ values at phase I show a tendency to rapidly decrease again. The similar tendency of the two ^1^H and ^13^C T_1ρ_ values means that ^1^H and ^14^N around ^13^C are bonded, and ^13^C and ^14^N around ^1^H are bonded, so it is thought that they do the same motions. And, the fact that the ^13^C T_1ρ_ value is longer than the ^1^H T_1ρ_ value means that the energy transfer of ^1^H bonded to the end of ^13^C is easy. Near T_C1_, T_C2_, and T_C4_, the T_1ρ_ values for ^1^H and ^13^C are more or less continuous, but T_1ρ_ values near T_C3_ are change discontinuous. To determine the magnitude of E_a_ depending the molecular dynamics, the logarithmic scale of T_1ρ_ values vs. 1000/T are shown as the solid lines in Fig. 8; E_a_ for ^1^H and was found to be 10.16 ± 0.88 kJ/mol at phases V and IV. And, the E_a_ for ^1^H was 37.08 ± 2.21 kJ/mol at phase I. On the other hand, the E_a_ for ^13^C obtained from the slope of T_1ρ_ as the function of inverse temperature at phases V and IV was 9.80 ± 0.45 and 9.83 ± 0.42 kJ/mol, whereas it for ^13^C was 45.04 ± 1.99 kJ/mol at phase I. Although the ^13^C chemical shifts are slightly different, E_a_ at phases V and IV is almost the same within the error range. It is noteworthy that the differences in E_a_ at phases V, IV, and I are very large.

The ^1^H and ^13^C T_1ρ_ values shows a tendency to increase rapidly at phases V and IV, whereas those shows decrease abruptly at phase I. The behaviours of the T_1ρ_ for Arrhenius-type molecular motions are separate into fast- and slow-motion parts. Fast motion is expressed as ω_1_τ_C_ << 1, T_1ρ_^–1^~ exp(E_a_/k_B_T), and the slow motion as ω_1_τ_C_ >> 1, T_1ρ_~ω_1_^–2^ exp(–E_a_/k_B_T)^38^. At the boundary of 300 K, it is divided into fast and slow motion. The ^1^H and ^13^C T_1ρ_ values at phases V and IV were in the fast-motion regime, whereas the ^1^H and ^13^C T_1ρ_ values at phase I were attributed to the slow-motion regime.

## Conclusion

Investigating of the growth, phase transition temperatures, and thermodynamics of the organic–inorganic hybrid [NH(CH_3_)_3_]_2_ZnCl_4_ crystals were considered. The orthorhombic structure of this crystal was determined by SCXRD, and the four phase transition temperatures of 257 K (= T_C4_), 286 K (= T_C3_), 326 K (= T_C2_), and 348 K (= T_C1_) were defined using DSC and PXRD results. The previously reported^19,34,35^ phase transition temperatures may slightly differ depending on the crystal growth conditions. This crystal had the thermal stability of about 495 K, and weight loss resulting in the loss of the HCl and 2HCl moieties was observed with increasing temperature owing to thermal decomposition. From the chemical shifts caused by the local field around ^1^H, the environments around ^1^H for CH_3_ does not changes according to the temperature change, whereas the environments around ^1^H for NH was changes. And, the coordination geometry of ^13^C becomes high symmetry as the temperature rises. As a result, the change in ^1^H chemical shifts around NH in the cation is suggest be due to the change in the hydrogen bond N‒H···Cl, which is related to the change in Cl around Zn in the anion. Finally, ^1^H T_1ρ_ and ^13^C T_1ρ_ values, which represent the energy transfer for the ^1^H and ^13^C atoms of the cation are changed significantly with temperature. The activation energies considered from the NMR T_1ρ_ values for molecular motion were very high at high temperature of phase I than at phase V and IV. Additionally, based on the relaxation time T_1ρ_ measurements in the rotational system for ^1^H and ^13^C at different temperatures, activation barriers for the molecular reorientation of the cation in phases I, IV, and V were determined. The activation energy barriers in phases IV and V are approximately four times lower than that in phase I. The relaxations show that in phases IV and V the energy barrier was related to the reorientation of methyl groups around the triple symmetry axis, and in phase I the reorientation of methyl groups of the cation was related to the entire rotation. This work provides an understanding of the fundamental properties for applications in organic–inorganic hybrid materials.

## Data Availability

The datasets generated and/or analysed during the current study are available in the CCDC 2321570.
